# Molecular Phylogenetic Analysis Reveals the New Genus *Hemisphaericaspora* of the Family Debaryomycetaceae

**DOI:** 10.1371/journal.pone.0103737

**Published:** 2014-07-30

**Authors:** Fengli Hui, Yongcheng Ren, Liang Chen, Ying Li, Lin Zhang, Qiuhong Niu

**Affiliations:** School of Life Science and Technology, Nanyang Normal University, Nanyang, PR China; The University of Hong Kong, Hong Kong

## Abstract

Four strains of a novel ascomycetous yeast species were recovered from the frass of wood-boring beetles collected from the Baotianman Nature Reserve and the Laojieling Nature Reserve in Henan Province, China. This species produced unconjugated and deliquescent asci with hemispheroid or helmet-shaped ascospores. Analysis of gene sequences for the D1/D2 domain of the large subunit (LSU) rRNA, as well as analysis of concatenated gene sequences for the nearly complete small subunit (SSU) rRNA and D1/D2 domain of the large subunit (LSU) rRNA placed the novel species in a small clade including only one recognised species, *Candida insectamans*, in the family Debaryomycetaceae (Saccharomycotina, Ascomycota). DNA sequence analyses demonstrated that the novel species was distinct from all currently recognised teleomorphic yeast genus. The name *Hemisphaericaspora nanyangensis* gen nov., sp. nov. is proposed to accommodate the novel genus and species. The new genus can be distinguished from closely related teleomorphic genera *Lodderomyces* and *Spathaspora* through sequence comparison and ascospore morphology. The ex-type strain of *H. nanyangensis* is CBS 13020^T^ ( = CICC 33021 = NYNU 13717). Furthermore, based on phenotypic and genotypic characteristics, *C. insectamans* is transferred to the newly described genus as *Hemisphaericaspora insectamans* comb. nov., in accordance with the changes in the International Code of Nomenclature for algae, fungi and plants.

## Introduction

The family Debaryomycetaceae was proposed by Kurtzman and Suzuki based on a concatenated dataset of gene sequences from D1/D2 large subunit (LSU) and small subunit (SSU) rRNA [1]. Currently, the family comprises 11 teleomorphic genera, namely *Debaryomyces*, *Kurtzmaniella*, *Lodderomyces*, *Meyerozyma*, *Millerozyma*, *Priceomyces*, *Scheffersomyces*, *Schwanniomyces*, *Spathaspora*, *Wickerhamia* and *Yamadazyma* [1], [2]. A number of anamorphic species within the family are currently members of the polyphyletic genus *Candida*. The majority of taxa included in the family *Debaryomycetaceae* form pseudohyphae, but species of the genus *Wickerhamia* do not [3]. With the exception of the genus *Spathaspora*, all teleomorphic species do not form septate hyphae [4]. Interestingly, most species of genus *Scheffersomyces* and *Spathaspora* possess the rare ability to ferment D-xylose, which gives them economic potential for production of bioethanol from plant waste residues [4]–[6].

At present, over 100 yeast species belong to the family Debaryomycetaceae. Members of the family are reported to have been isolated from a wide variety of substrates, such as soil, water, foods, plant substrates and animal-associated samples [7], [8]. Numerous of these yeast species are known to be associated with wood-feeding insects and specifically *Scheffersomyces* and *Spathaspora* members commonly found in the gut of this insects. For example *Pichia stipitis* (* = Scheffersomyces stipitis*)-like yeasts are reported to be obligately associated with wood-boring beetles [6]. Other insect associates are *Candida entomaea* (* = Yamadazyma mexicana*) from insect tunnels, *Spathaspora arborariae* from rotting wood and *Candida insectamans* from the frass of beetle larvae [9]–[11]. More recently, Suh et al. described four other insect-associated species, *Scheffersomyces parashehatae*, *Scheffersomyces xylosifermentans*, *Candida broadrunensis* and *Candida manassasensis*, isolated from wood-boring beetles, their frass and galleries [12].

In a study of yeasts from wood-boring insects, we isolated a large number of yeasts mainly from the digestive tract of beetles as well as from related substrates, including rotted wood, frass and galleries [13], [14]. The majority of the yeast belonged to several major clades in the Saccharomycotina; some of these species have been identified as novel species in earlier papers [13]–[16]. Amongst the insect associates, we focused on four strains of a sexual ascomycetous yeasts from the frass of beetle larvae in China. The strains produce hemispheroid or helmet-shaped ascospores in unconjugated and deliquescent asci. Analysis of the D1/D2 domain of the LSU rRNA gene sequence alone as well as a phylogenetic analysis based on the combined gene sequences of nearly the entire SSU and D1/D2 domain of the LSU rRNA revealed that the above-noted strains represent an undescribed teleomorphic yeast species in the *Candida insectamans* clade. This clade is phylogenetically well separated from currently recognised teleomorphic genera assigned to the family Debaryomycetaceae. Hence, we propose the new genus *Hemisphaericaspora* gen. nov. and the novel species *Hemisphaericaspora nanyangensis* gen. nov., sp. nov. to accommodate these strains.

## Materials and Methods

### Yeast Isolation and Culture

Frass samples of wood-boring beetles were collected in the Baotianman Nature Reserve (33°27′N and 111°48′E) and the Laojieling Nature Reserve (33°44′N and 111°28′E) located in Henan Province, China, at the Southern part of Funiu Mountain (Table 1). All necessary permits were obtained from Baotianman Nature Reserve Administration of Henan, China, and from Laojieling Nature Reserve Administration of Henan, China. The field collections were made according to Chinese diversity rules, and the field studies did not involve endangered or protected species. Isolation of yeast strains from insect frass was performed through a previously described method [17]. Samples were directly streaked on acidified yeast extract-malt extract (YM) agar (0.3% yeast extract, 0.3% malt extract, 0.5% peptone, 1% glucose, 2% plain agar, adjusted to pH 3.5 with HCl) plates supplemented with 0.025% sodium propionate and 200 mg chloramphenicol L^−1^. Cultures were incubated at 25°C, and single yeast colonies were streaked at least twice for purification. Purified yeast strains were maintained in YM slants or 30% (v/v) glycerol.

**Table 1:** Summary of new species isolated in this study.

Isolation No.	Strain designation^a^		Source	Locality
	CICC	CBS		
NYNU 13715	–	–	Frass from *Quercus aliena*	Baotianman Mountain, Henan, China
NYNU 13717	33021	13020^T^	Frass from *Quercus variabilis*	Baotianman Mountain, Henan, China
NYNU 13729	–	–	Frass from beetle larva	Baotianman Mountain, Henan, China
NYNU 13745	–	–	Frass from *Quercus aliena*	Laojieling Mountain, Henan, China

a T, ex-type strain; CICC, China Center of Industrial Culture Collection, Beijing, China; CBS, Centraalbureau voor Schimmelcultures, Utrecht, The Netherlands.

### Morphological, Physiological and Biochemical Characteristics

The standard yeast description includes morphological observations and metabolic tests performed according to established methods [18], [19]. Assimilation tests for carbon and nitrogen sources were performed in liquid media. Starved inocula were used in nitrogen and vitamin assimilation tests. Growth at various temperatures was determined by cultivation on YM agar. Mating and ascospore formation were investigated by growing individual or mixed strain pairs on YM agar, 5% malt extract agar, cornmeal agar and yeast carbon base supplemented with 0.01% ammonium sulfate (YCBS) agar at 17 and 25°C for 1–4 weeks.

### DNA Extraction, PCR Amplification and Sequencing

Genomic DNA was extracted with a Dr. GenTLE (from yeast) High Recovery kit according to the manufacturer's protocol (Takara Bio, Shiga, Japan). The concentration, integrity and purity of total extracted DNA were confirmed by gel electrophoresis in 0.8% agarose in 0.5× Tris-Borate-EDTA (TBE). The D1/D2 domain of the LSU rRNA gene and internal transcribed spacer (ITS) region were amplified by PCR and sequenced using the primer pairs NL1 and NL4 [20] and ITS1 and ITS4 [21], respectively. The nearly complete SSU rRNA gene sequence was determined using a method described by Kurtzman and Robnett [22]. PCR conditions recommended in the references for each primer pair were used. The purified PCR products were sequenced using a Dye Terminator cycle sequencing kit (Applied Biosystems, Warrington). Sequences determined in this study were deposited in the GenBank database. The GenBank/EMBL/DDBJ accession numbers for the sequences of isolate CBS 13020^T^ (NYNU 113717) are KF690375 (D1/D1 domain of the LSU rRNA gene), KF690366 (ITS) and KF690370 (SSU rRNA gene).

### Phylogenetic Analysis

**Figure 1:**
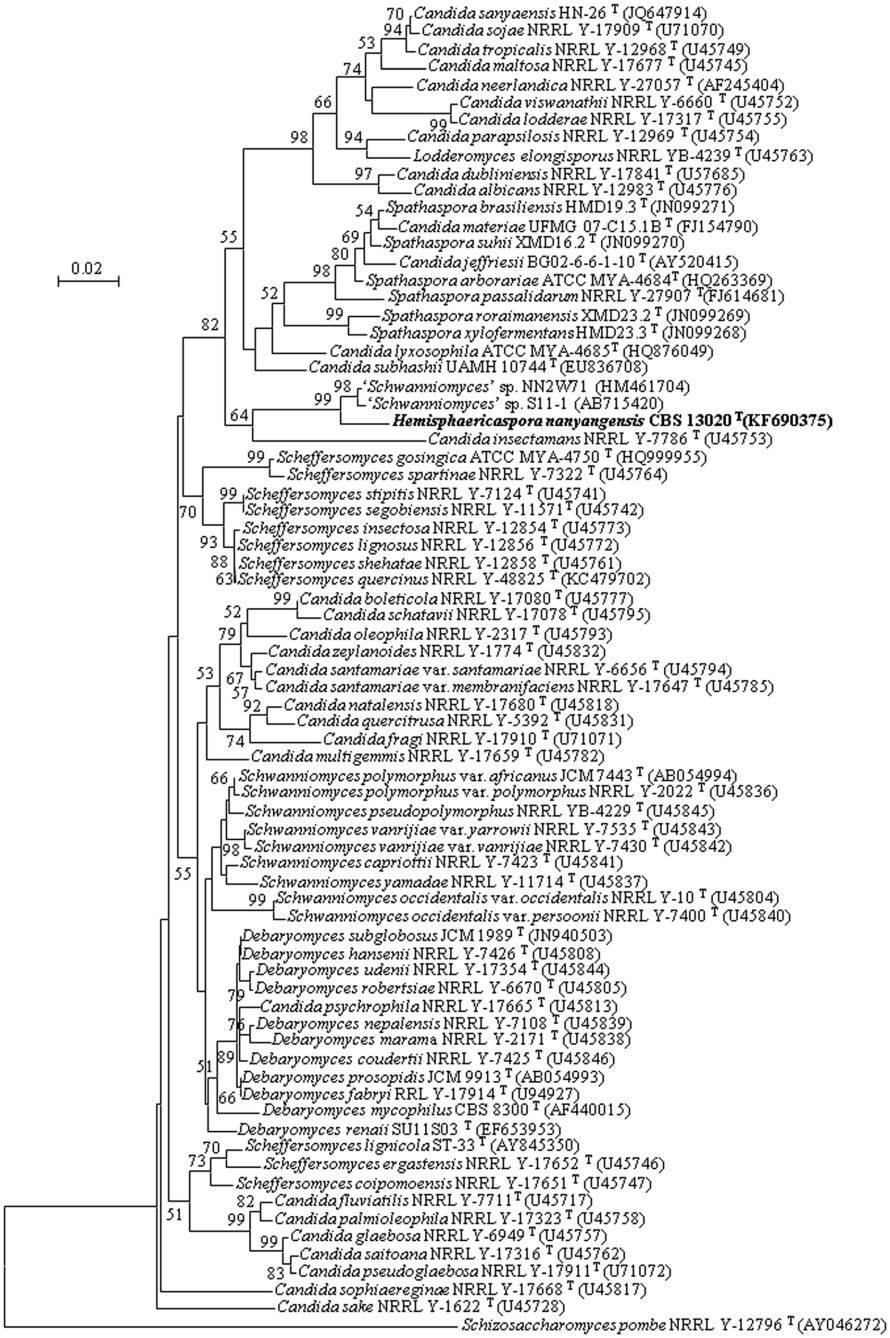
Phylogenetic tree constructed by neighbour-joining analysis based on the sequence of the D1/D2 domain of the large subunit rRNA gene showing the relationships of *Hemisphaericaspora nanyangensis* sp. nov. and other species of the genera assigned to Debaryomycetaceae [1], [12], [33], [34]. Bootstrap values above 50% are given at nodes based on 1,000 replications. Bar, 0.02 substitutions per nucleotide position. *Schizosaccharomyces pombe* NRRL Y-12796^T^ was used as an outgroup.

**Figure 2:**
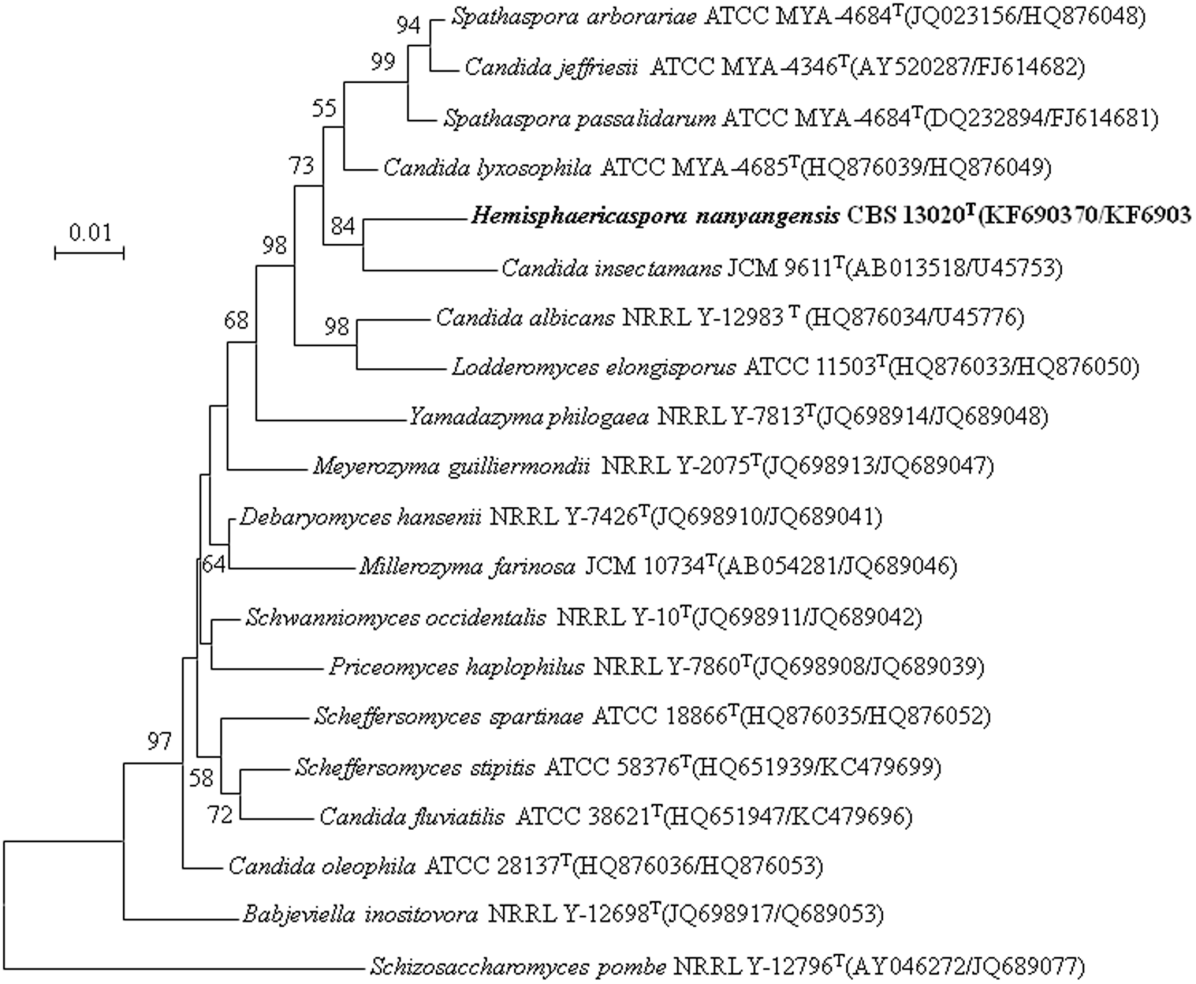
Phylogenetic tree constructed by maximum likelihood analysis based on the combined gene sequences of the nearly entire SSU rRNA and D1/D2 domain of LSU rRNA depicting the placement of *Hemisphaericaspora nanyangensis* sp. nov. and representative neighbouring taxa in the family Debaryomycetaceae [2], [12], [30]. Bootstrap values above 50% are given at nodes based on 1,000 replications. The sequence accession numbers are shown in parentheses with the first number representing SSU rRNA gene and the second representing D1/D2 LSU rRNA gene. Bar, 0.01 substitutions per nucleotide position. *Schizosaccharomyces pombe* NRRL Y-12796^T^ was used as an outgroup.

The sequences were compared pairwise through a BLASTN search [23] and aligned with sequences of related species retrieved from GenBank using the multiple alignment program CLUSTAL X version 1.83 [24]. Phylogenetic trees were constructed by the neighbour-joining method [25] with the evolutionary distance data calculated from Kimura's two-parameter correction [26] and the maximum likelihood method using the Tamura-Nei model [27] of MEGA version 5 [28]. Sites containing gaps in the alignments of a single gene or combined sequences were excluded. Confidences for the phylogenetic tree were estimated from bootstrap analysis (1000 replicates). The reference sequences used in this paper were retrieved from GenBank under the accession numbers indicated in Fig. 1 and 2.

### Nomenclature

The electronic version of this article in Portable Document Format (PDF) in a work with an ISSN or ISBN will represent a published work according to the International Code of Nomenclature for algae, fungi, and plants, and hence the new names contained in the electronic publication of a PLOS ONE article are effectively published under that Code from the electronic edition alone, so there is no longer any need to provide printed copies.

In addition, new names contained in this work have been submitted to MycoBank from where they will be made available to the Global Names Index. The unique MycoBank number can be resolved and the associated information viewed through any standard web browser by appending the MycoBank number contained in this publication to the prefix http://www.mycobank.org/MB. The online version of this work is archived and available from the following digital repositories: PubMed Central; LOCKSS.

## Results and Discussion

### *Hemisphaericaspora nanyangensis* gen. nov., sp. nov., an Ascosporogenous Yeast Species

The four isolates of *Hemisphaericaspora nanyangensis* were identical in sequences, morphology, and physiology. BLAST sequence similarity search of the GenBank database showed no other yeast taxon to have a D1/D2 sequence identical to the novel species. The closest matches, in terms of D1/D2 sequence similarity, were two undescribed ‘*Schwanniomyces*’ species namely strains NN2W71 and S11-1 (HM461704 and AB715420), with over 2% sequence divergence (12 substitutions). The new species significantly differed from its nearest phylogenetic neighbour, *Candida insectamans* (U45753), by 12.6% sequence divergence (63 substitutions and 9 gaps) in D1/D2 sequences. For the ITS region, this new species differed by 7.6% sequence divergence (24 substitutions and 11 gaps) from their closest relative, the unidentified ‘*Schwanniomyces*’ sp. strain NN2W71 (JQ901901), but the pairwise sequence analysis with ‘*Schwanniomyces*’ sp. strain S11-1 and *C. insectamans* could not be performed because their ITS sequences are not currently available from either the NCBI GenBank database or from the CBS database. According to the yeast species recognition from molecular data discussed in Kurtzman and Robnett and Kurtzman [20], [29], we concluded that the new isolates represent a novel species distinct from presently described and undescribed species.

The phylogenetic placement of the novel species based on D1/D2 sequences is shown in Fig. 1. D1/D2 sequence analysis indicates that *H. nanyangensis* sp. nov. is genetically separate from known species and forms a small isolated clade along with two undescribed yeast strains and *Candida insectamans*. This clade is related to the *Lodderomyces* and *Spathaspora* clades. As D1/D2 sequence analysis seldom provides strong support for deep lineages, the hypothesis that *H. nanyangensis* sp. nov. represents a separate genus was tested by analysing concatenated gene sequences from nearly the entire SSU rRNA and D1/D2 domain of the LSU rRNA. Reference species included representatives of neighbouring teleomorphic genera and phylogenetically isolated neighbouring *Candida* lineages assigned to the family Debaryomycetaceae [2], [12], [30]. Maximum likelihood analysis showed that the proposed genus *Hemisphaericaspora* gen. nov. is well separated from neighbouring species and genera (Fig. 2). In the analysis, the *Hemisphaericaspora* clade (84% bootstrap support) is flanked by sister clades. From the preceding analysis, the *Hemisphaericaspora* clade appears to be phylogenetically separate from other clades of the family Debaryomycetaceae, which supports the proposal that *H. nanyangensis* sp. nov. represents a new genus. Moreover, two undescribed ‘*Schwanniomyces*’ species that share similar D1/D2 sequences (2 bp difference) cluster near the ex-type strain of *H. nanyangensis* sp. nov. (Fig. 1), which suggests that they represent a novel species of the new genus proposed in this study but not a new *Schwanniomyces* species.

The new genus for *Hemisphaericaspora* could be recognised from neighbouring sister genera *Lodderomyces* and *Spathaspora* by the morphology of ascospores. *L. elongisporus* and species of the genus *Spathaspora* are known to produce ellipsoidal or elongated ascospores [4], [31], whereas *H. nanyangensis* sp. nov. has hemispheroid or helmet-shaped ascospores. *H. nanyangensis* sp. nov. could be further distinguished from its phylogenetically closest recognised neighbour, *C. insectamans*, based on the following characteristics: *H. nanyangensis* sp. nov. assimilates sucrose, raffinose, D-gluconate and DL-lactate but unable for soluble starch, succinate and citrate. *H. nanyangensis* sp. nov. possesses the ability to grow in vitamin-free medium and 1% acetic acid and at 42°C. *H. nanyangensis* sp. nov. lacks the ability to ferment glucose (Table 2). Although *H. nanyangensis* sp. nov. can be distinguished from *C. insectamans* by standard phenotypic characteristics, DNA sequence-based identification is desirable because phenotype-based identification is potentially time-consuming, expensive and inaccurate.

**Table 2:** Physiological characteristics that differentiate *H. nanyangensis* sp. nov. from *C. insectamans* a.

Characteristic	*H. nanyangensis*	*C. insectamans* b
Fermentation of		
Glucose	−	s
Assimilation of		
Sucrose	w	−
Raffinose	**+**	−
Soluble starch	−	**+**
D-Gluconate	w	−
DL-Lactate	**+**	−
Succinate	−	**+**
Citrate	−	**+**
Ethanol	−	**+**
Growth in/at		
Acetic acid 1%	**+**	−
Vitamin-free medium	**+**	−
42°C	**+**	−

a +, Positive; −, negative; s, slowly positive; and w, weakly positive.

b Data from [35].

### Phylogenetic Placement of *Candida insectamans*

*C. insectamans* was described to accommodate an isolate from the frass of buprestid beetle larvae in southern Africa [11]. Kurtzman and Robnett linked the species to *Candida sake* on the basis of the D1/D2 domain of LSU rRNA gene sequences [20]. Analysis of available SSU rRNA gene dates weakly links *C. insectamans* to *Candida lyxosophila* and to the *Lodderomyces* clade [32]. Studies based on both sequences document a link to the ascogenous genus *Spathaspora* and related *Candida* species, including *C. lyxosophila* [1],[4]. *C. insectamans* formed a genetically separate lineage within the *Spathaspora* clade but with little or no bootstrap support in the D1/D2 tree drawn by Cadete et al [33]. Phylogenetic analysis based on the D1/D2 domain of the LSU rRNA gene in this study indicated that *C. insectamans* and *H. nanyangensis* sp. nov. are related (Fig. 1). This finding was supported with high bootstrap values and confirmed by the phylogenetic tree based on the combined gene sequences of nearly the entire SSU rRNA and D1/D2 domain of LSU rRNA (Fig. 2). Nucleotide divergence in the SSU rRNA gene is concordant with that in the D1/D2 domain of the LSU rRNA gene and further suggests that *C. insectamans* and *H. nanyangensis* sp. nov. are closely related to each other. Furthermore, some morphological characteristics of *C. insectamans*, such as white, glossy colonies with filamentous margin, are somewhat similar to those of the new species. Based on our phylogenetic analysis and the phenotypic characteristics we observed, we suggest that *C. insectamans* should be transferred to the genus *Hemisphaericaspora* and propose a new combination.

### Description of *Hemisphaericaspora* Hui, Ren, Chen, Li, Zhang & Niu gen. nov

Hui et al. 2014, sp. nov. [urn:lsid:imycobank.org:names: MycoBank accession number MB 808357.

Asexual reproduction is by budding. Vegetative cells are mostly globose or subglobose. Septate true hyphae and pseudohyphae are present. Globose or subglobose asci arise without conjugation and contain one to two hemispheroid or helmet-shaped ascospore. Asci are deliquescent. The single known species does not ferment sugars. Nitrate is not utilized as a sole source of nitrogen. Acetic acid is not produced. Extracellular starch is not produced. Diazonium blue B reaction is negative. The genus *Hemisphaericaspora* appears most closely related to the genera *Lodderomyces* and *Spathaspora* (Fig. 1 and 2). The new genus can be separated from other members of the Debaryomycetaceae by gene sequence analysis.

#### Type species

*Hemisphaericaspora nanyangensis* Hui, Ren, Chen, Li, Zhang & Niu.

#### Etymology

The generic name *Hemisphaericaspora* (He.mis.phae.rica'spo.ra; Lat., adj. fem.) refers to the hemisphere shape of the ascospores produce by type culture of *Hemisphaericapora nayangensis*.

### Description of *Hemisphaericaspora nanyangensis* Hui, Ren, Chen, Li, Zhang & Niu sp. nov

Hui et al. 2014, sp. nov. [urn:lsid:imycobank.org:names: MycoBank accession number MB 808358.

**Figure 3:**
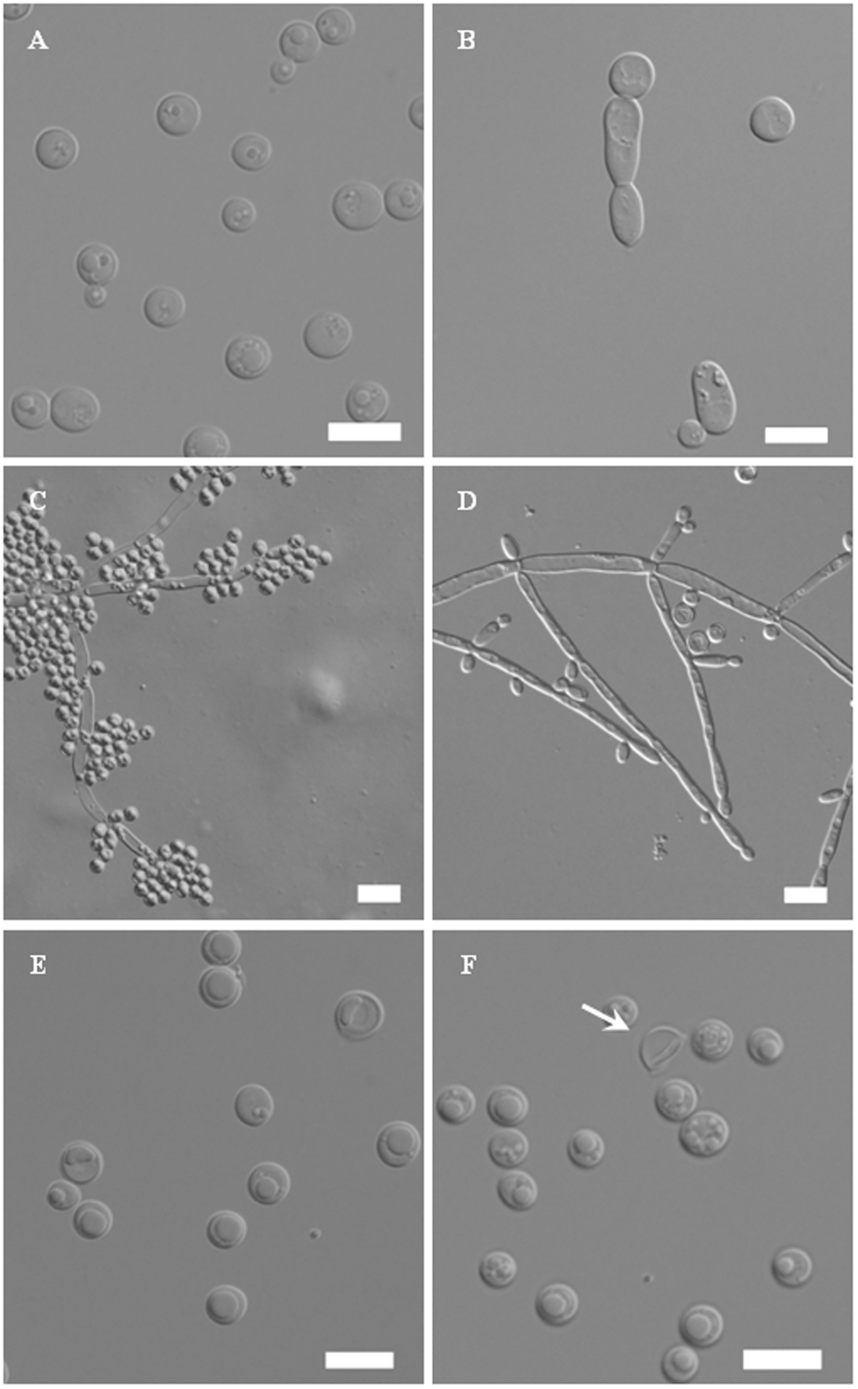
Photomicrographs of *Hemisphaericaspora nanyangensis* sp. nov. CBS 13020^T^. (A) Budding cells on YM broth after 3 days at 25°C. (B) Elongated vegetative cells on YM cultured for 7 days at 25°C. (C) Pseudohyphae on cornmeal agar after 12 days at 25°C. (D) Septate hyphae with vegetative cells on cornmeal agar after 12 days at 25°C. (E) An ascospore within the ascus after 1 week on YCBS agar at 25°C. (F) An ascospore (arrow) released from the ascus after 2 week on YCBS agar at 25°C. Bar = 10 µm.

After 3 days at 25°C in YM broth, cells are globose to subglobose (3–8×4–10 µm), occurring singly or in pairs (Fig. 3A). Budding is multilateral. Septate hyphae may be present but no pseudohyphae are observed. Sediment is formed after a month but a pellicle is not observed. After 7 days at 25°C on YM agar, elongated vegetative cells are present (Fig. 3B). Colonies are white, butyrous, smooth and glistening. Colony margins are mostly smooth with occasional filamentous growth. After 12 days at 25°C on Dalmau plate culture on cornmeal agar, pseudohyphae and septate hyphae with vegetative cells are formed (Fig. 3C–D). Aerobic growth is white, dull and wrinkled with a fringed margin. Asci are globose or subglobose and commonly from single cell on cornmeal agar and YCBS agar at 25°C after 6 days (Figs. 3E). Each ascus contains one to two hemispheroid or helmet-shaped ascospores. Asci are deliquescent (Figs. 3F). Results of fermentation and assimilation tests commonly used in yeast taxonomy are given in Table 3.

**Table 3:** Physiological characteristics and other growth tests of *H. nanyangensis* sp. nov.^a^

Fermentation					
D-Glucose	−	Raffinose	−	Melibiose	−
D-Galactose	−	α,α-Trehalose	−	Melezitose	−
Sucrose	−	Inulin	−	Starch	−
Maltose	−	Cellobiose	−	D-Xylose	−
Lactose	−	Methyl-α-D-glucoside	−		
Assimilation of carbon compounds					
D-Glucose	**+**	Salicin	**+**	D-Mannitol	**+**
D-Galactose	−	Arbutin	−	Galactitol	−
L-Sorbose	−	Melibiose	−	*myo*-Inositol	−
D-Glucosamine	−	Lactose	−	D-Glucono-1,5-lactone	−
D-Ribose	w	Raffinose	**+**	2-Keto-D-Gluconate	w
D-Xylose	w	Melezitose	w	5-Keto-D-Gluconate	**+**
L-Arabinose	−	Inulin	−	D-Gluconate	w
D-Arabinose	−	Soluble starch	−	D-Glucuronate	−
L-Rhamnose	−	Glycerol	w	D-Galacturonic acid	**+**
Sucrose	w	Erythritol	−	DL-Lactate	**+**
Maltose	**+**	Ribitol	**+**	Succinate	−
α,α-Trehalose	**+**	Xylitol	−	Citrate	−
Methyl-α-D-glucoside	**+**	L-Arabinitol	−	Methanol	−
Cellobiose	**+**	D-Glucitol	−	Ethanol	−
Assimilation of nitrogen compounds					
Nitrate	−	Cadaverine	−	Imidazole	**+**
Nitrite	−	Creatine	−	D-Tryptophan	−
Ethylamine	**+**	Creatinine	−		
L-Lysine	**+**	Glucosamine	−		
Growth tests					
10%NaCl/5% glucose	−	0.01% Cycloheximide	−	Growth at 37°C	**+**
16%NaCl/5%glucose	−	0.1% Cycloheximide	−	Growth at 40°C	**+**
50% Glucose	−	Vitamin-free medium	**+**	Growth at 42°C	**+**
60% Glucose	−	1% Acetic acid	**+**	Growth at 45°C	−
Additional tests					
Starch formation	−	Urea hydrolysis	−		
Acetic acid production	−	Diazonium blue B reaction	−		

a +, Positive; −, negative; and w, weakly positive.

#### Ex-type strain

CBS 13020^T^ ( = CICC 33021) is preserved as a lyophilised preparation at the China Center of Industrial Culture Collection (CICC), Beijing, China, as well as at the Yeast Collection of Centraalbureau voor Schimmelcultures (CBS), Utrecht, the Netherlands. The strain was isolated from the frass of beetle larvae collected in July 2013 from Baotianman Nature Reserve in Nanyang, Henan Province, central China (Table 1), the coordinates for which are 33°27′N and 111°48′E.

#### Etymology

The species epithet *nanyangensis* (nan.yang.en'sis. L. nom. masc. adj.) refers to Nanyang, Henan Province, central China, the geographical origin of the species.

### New Species Combinations

*Hemisphaericaspora insectamans* (D. B. Scott, van der Walt & Klift) Hui, Ren, Chen, Li, Zhang & Niu comb. nov.

Basionym: *Candida insectamans* D. B. Scott, van der Walt & Klift Mycopathologia 47: 227, 1972.

Ex-type strain: CBS 6033^T^ ( = NRRL Y-7786).

MycoBank accession number: MB 808359.
